# Spread of the Optical Power Emission of Three Units Each of Two Different Laser Therapy Devices Used in Sports Medicine, Which Cannot Be Assessed by the Users, Shown by Means of High-Fidelity Laser Measurement Technology

**DOI:** 10.3390/biomedicines11020585

**Published:** 2023-02-16

**Authors:** Leon Kaub, Christoph Schmitz

**Affiliations:** Department of Anatomy II, Faculty of Medicine, LMU Munich, 80336 Munich, Germany

**Keywords:** laser beam characterization, laser therapy, medical devices, sports medicine

## Abstract

Laser therapy devices (LTDs) operating with near-infrared laser light are increasingly being used in sports medicine. For several reasons, users cannot evaluate whether or not such devices emit laser beams according to the specifications provided by the manufacturer and the settings of the device. In this study, the laser beams from two different LTDs that can be used in sports medicine were thoroughly characterized by measuring the emitted power, pulse shapes and lengths and spatial intensity distributions using professional, high-fidelity laser measurement technology. This was repeated for three units of each LDT independently to distinguish problems of individual units from potential intrinsic instrument design errors. The laser beams from the units of one LTD agreed with the settings of the device, with the measured average power for these units being within 3.3% of the set power. In contrast, the laser beams from the units of the other LTD showed large deviations between the settings and the actual emitted light. This device came with three laser diodes that could be used independently and simultaneously. The average power differed greatly between the units as well as between the laser diodes within each unit. Some laser diodes emitted essentially no light, which could lead to a lack of treatment for patients. Other laser diodes emitted much more power than set at the device (up to 230%), which could result in skin irritations or burning of patients. These findings indicate a need for better standardization and consistency of therapeutic laser light sources.

## 1. Introduction

Laser therapy devices (LTDs) operating with near-infrared laser light are increasingly being used in sports medicine and sports physiotherapy to treat, e.g., Achilles tendinopathy [1], carpal tunnel syndrome [2], rotator cuff tendinopathy [3], structural muscle injury [4] and exercise-induced skeletal muscle fatigue [5], to mention only a few indications (see also [6]). For several reasons, users (physicians and physiotherapists) cannot evaluate whether or not such devices emit laser beams according to the specifications provided by the manufacturer and the settings of the device. Among these reasons is the fact that infrared light is invisible to the human eye. Furthermore, a thorough assessment of the characteristics of the laser beam of an LTD, which comprises the emitted power, pulse shapes and lengths, as well as spatial intensity distributions, must be performed using professional, high-fidelity laser measurement equipment whose operation requires expert knowledge at the level of an engineer or a physicist [7,8].

It is, therefore, all the more important that the set and the actual values of LTDs actually match. An LTD that emits much less power than set at the device results in a lack of treatment, although the therapist and the patient think differently. In contrast, an LTD that emits much more power than set at the device might result in undesired effects such as skin irritations or burnings of the patient. In addition, the use of malfunctioning LTDs can lead to false negative results in clinical studies, in which the actual light emission of the used LTD can usually not be assessed by the practitioner. Considering the worldwide, strict regulation of medical devices (in Europe, the Medical Device Directive 93/42/EEC and the new Medical Device Regulation 2017/745), one would expect that differences between the set and the actually emitted optical power emission of an LTD should be small and within the range that is specified in the user manual.

During the course of a recent study, we investigated the penetration depth of near-infrared laser light into biological tissue emitted by two different LTDs [7]. When performing the experiments for this study, we realized that one of the used LTDs showed large deviations between the set and the actual measured power [7]. In order to see whether this behavior was due to a single broken laser diode, we measured two more units of the same LTD. In addition to the average power, further laser beam characteristics such as the pulse shape, pulse length and spatial intensity distributions were investigated. To compare the findings from the three units of this LTD, we also measured the beam parameters of three units of the other LTD that was used in the previous study [7]. All tested units of the LTDs were in clinical use before they were characterized in the present study. Furthermore, both LTDs were commercially available at the time when the present study was performed (Fall 2022 and Winter 2022/23). It was our hypothesis that the large deviations between the set and the actual measured power of one unit of the first LTD observed during our recent study was an isolated case.

All measurements for the present study were performed in a laboratory environment using professional, high-fidelity laser measurement equipment.

## 2. Materials and Methods

**Figure 1:**
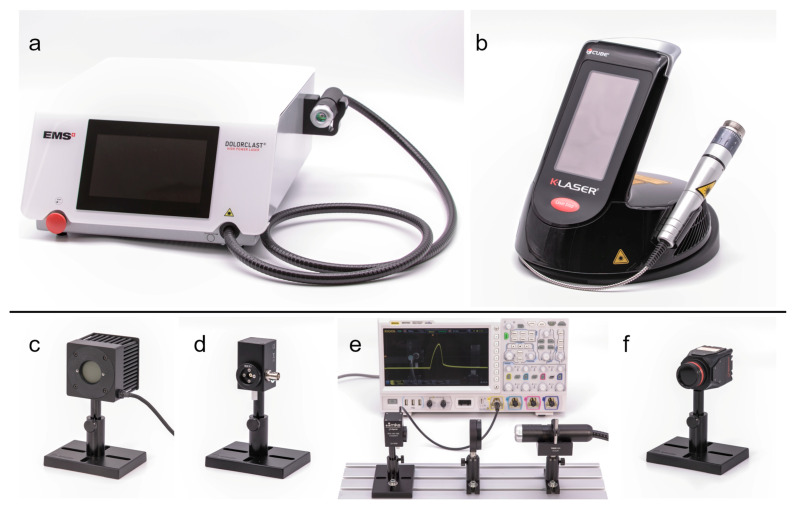
Laser therapy devices and sensors used in the present study to investigate laser beam parameters: (**a**) EMS laser; (**b**) K-laser; (**c**) thermal power sensor; (**d**) photodiode; (**e**) experimental setup to measure temporal profiles showing the diffusor in between the photodiode and the handpiece of the EMS laser as well as the oscilloscope (MSO7024) that recorded signals from the photodiode; (**f**) beam profiling camera. Modified from [7] with permission from the authors. Details are in the text.

Three separate units of the Dolorclast High Power Laser (Electro Medical Systems, Nyon, Switzerland; hereafter: EMS lasers and EMS-1, EMS-2 and EMS-3, Figure 1a) and 3 separate units of the Cube 4 Med (Eltech K-Laser s.r.l., Treviso, Italy; hereafter: K-lasers and K-1, K-2 and K-3; Figure 1b) were investigated. The LTDs were made available by physicians and physiotherapists from different clinics in Germany and Australia and had been in therapeutic use before investigation. The serial number of all units was recorded. The units of each manufacturer had the same operating software version installed (EMS lasers, EMS medical devices version 1.0, Electro Medical Systems; K-lasers, CUBE 4 Software Version 2, Eltech K-Laser). The handpieces of the LTDs came with a spacer that was removed for all measurements. All LTDs and sensors were cleaned with professional cleaning equipment for optical components before each measurement.

The EMS lasers operated with 1 wavelength (905 nm) and in pulsed wave (PW) mode only. The repetition rate was the only parameter that could be set. It was varied between 5 and 40 kHz for measurements in the present study; larger repetition rates up to 80 kHz would have been possible only with a modulation lower than 100% (i.e., no continuous repeat of pulses in time) and were therefore not used in the present study. According to the user manual, the repetition rate translates directly to average power since the device was designed to always emit light pulses with the same amplitude and length. Therefore, the maximum average power with 40 kHz was 1.2 W.

Each K-laser came with the same set of 4 different laser diodes and therefore allowed to select different wavelengths (660 nm, 800 nm, 905 nm and 970 nm) as well as different operating powers (1–12 W) and, respectively, the continuous wave (CW) mode or PW mode with different repetition rates (1 Hz–20 kHz). It was also possible to operate the K-lasers with all 4 diodes simultaneously in a normal mode and in a mode called intense super pulse (ISP). The 660 nm diode of the K-lasers was not measured individually since the maximum power of this diode was only 0.1 W (c.f. [7]).

Three different sensors were used to measure the power, temporal characteristics and spatial intensity distributions of the laser beams. Table 1 summarizes the selected sensitivity measures of the sensors used in the present study.

The average power was measured with a thermal power sensor (Model 50(150)A-BB-26-PPS; Ophir Spiricon Europe GmbH, Darmstadt, Germany) that had a noise level of 2 mW (Figure 1c). This sensor had the ability to measure beam positions and widths, which was used to center the laser beams during the experiments and to ensure that the laser beams had the same beam width for all power measurements (8 mm). The power of each device and mode was measured at multiple different average powers set at the device interfaces: EMS laser, 8 steps from 0.15 W to 1.2 W; K-laser CW mode and ISP mode, 12 steps from 1 W to 12 W; K-laser PW mode, 12 steps from 0.5 W to 6 W. At each step, 10 measurements were recorded and averaged. EMS-1 was only measured at 3 steps (0.15 W, 0.6 W and 1.2 W), with each step being recorded for 3 min. For the K-lasers, the measurements were repeated for each unit using its 5 different modes (800 nm, 905 nm, 970 nm, all diodes and ISP) and 7 different repetition rates (CW, 1 Hz, 10 Hz, 100 Hz, 10 kHz, 10 kHz and 20 kHz), except for the ISP mode that could not be operated in CW mode.

Temporal characteristics of the laser pulses were measured with a photodiode (FPD-VIS300, Ophir Spiricon Europe GmbH; hereafter: FPD; Figure 1d). The FPD was connected to an oscilloscope (MSO7024; Rigol Technologies, Inc., Suzhou, China) that recorded signals with a sampling rate of 10 Gigasamples per second. An optical diffusor (DG20-220-MD, Thorlabs GmbH, Bergkirchen, Germany) in the beam line protected the FPD from large power densities (Figure 1e). The recordings were made with the same distances between the laser, diffusor and sensor. EMS-1 and K-2 were recorded with different distances and different oscilloscopes (HMO3524, Hameg Instruments GmbH, Mainhausen, Germany). The EMS lasers were measured at 5 repetition rates (5 kHz, 10 kHz, 20 kHz, 30 kHz and 40 kHz) except for EMS-1 (only 5 kHz, 20 kHz and 40 kHz). The K-lasers were recorded at 6 repetition rates (1 Hz, 10 Hz, 100 Hz, 1 kHz, 10 kHz and 20 kHz) and with the LTDs set to an average power of 6 W (normal modes) or 12 W (ISP mode).

Spatial light intensity distributions were recorded with a beam profiling camera (LT665; Ophir Spiricon Europe GmbH) (Figure 1f) that was operated with software from the manufacturer (BeamGage Professional v6.17.1; Ophir Spiricon Europe GmbH). The CCD sensor of this camera had an active area of 1.25 cm² (12.5 × 10 mm). The distance between the laser handpieces and the camera was 3 mm. The camera exposure time was adapted to maximize the recorded intensities. The laser beams were operated with low average powers and in CW or at the lowest repetition rate (EMS laser, 0.15 W, 5 kHz; K-laser, 0.5 W, CW mode). In some cases, the average power had to be adjusted to ensure that the laser beams had a sufficiently high intensity. The K-lasers came with a zoom objective at their handpiece, which allowed recording the laser beams at different beam sizes. The scale at the handpiece ranged from 1 to 5, which according to the manufacturer, translates to a beam area of 1–5 cm² at the distance that is given by the handpiece’s spacer.

## 3. Results

### 3.1. EMS Laser

**Figure 2:**
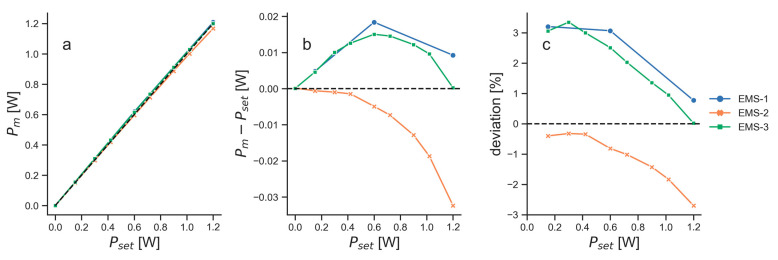
Power measurements of the three EMS lasers (EMS-1, blue dots and blue lines; EMS-2, orange dots and orange lines; EMS-3, green dots and green lines): (**a**) measured power ($P_{m}$) for eight different powers set at the EMS laser units ($P_{set}$); (**b**) the difference between $P_{m}$ and $P_{set}$ at each set power; (**c**) deviation of $P_{m}$ compared to $P_{set}$ as a percentage. Black dashed lines illustrate ideal curves where $P_{m}$ equals $P_{set}$.

The measured power ($P_{m}$) of EMS-1, EMS-2 and EMS-3 showed, in general, good agreement with the power that was set for the devices ($P_{set};$ Figure 2). EMS-1 and EMS-3 tended to emit more power than set, with a maximum deviation of 18 mW (EMS-1) and 15 mW (EMS-3) at $P_{set}$ = 0.6 W (i.e., the relative deviation was 3% in the case of EMS-1 and 2.5% in the case of EMS-3). In contrast, EMS-2 emitted increasingly less power for higher values of $P_{set}$ with a maximum deviation of 32 mW at $P_{set}$ = 1.2 W (relative deviation, 2.7%).

**Figure 3:**
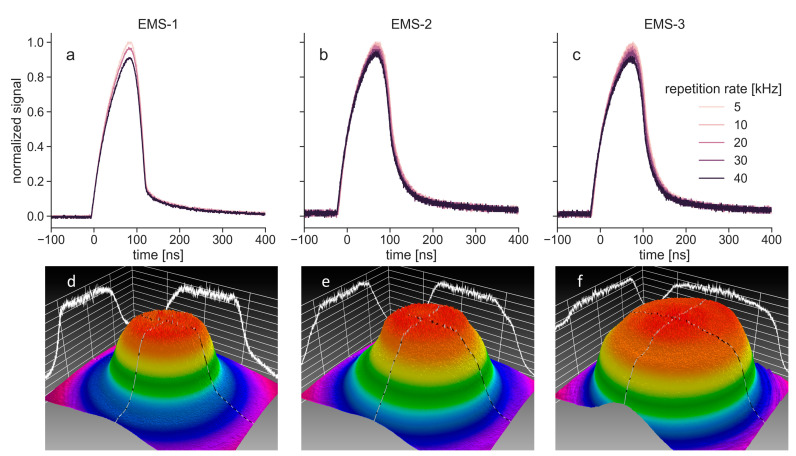
Temporal and spatial characteristics of the three EMS lasers (EMS-1, EMS-2 and EMS-3): (**a**–**c**) pulse recordings of each EMS laser recorded at different repetition rates. EMS-1 was measured at three repetition rates while EMS-2 and EMS-3 were also measured at 10 kHz and 30 kHz; (**d**–**f**) color-coded spatial intensity distributions of the three EMS lasers. Magenta represents the lowest light intensity, and red represents the highest light intensity. White lines that are projected onto the grid planes are intensity profiles along center lines in the x- and y-directions.

Figure 3a–c shows the light intensity as a function of time for the light pulses of the EMS lasers at different repetition rates. The signals were normalized to the maximum value, which was measured at 5 kHz for all three units. With increasing repetition rate, the pulse amplitude of the three units decreased to approximately 90% of the maximum at 40 kHz. Other than this decrease in amplitude, there were no differences in pulse shape and length. Camera images of the EMS lasers (Figure 3d–f) show that all three devices had an almost ideal flat-top beam shape (c.f. [7]), although the beam width varied between the three devices.

### 3.2. K-Laser Power Measurements

**Figure 4:**
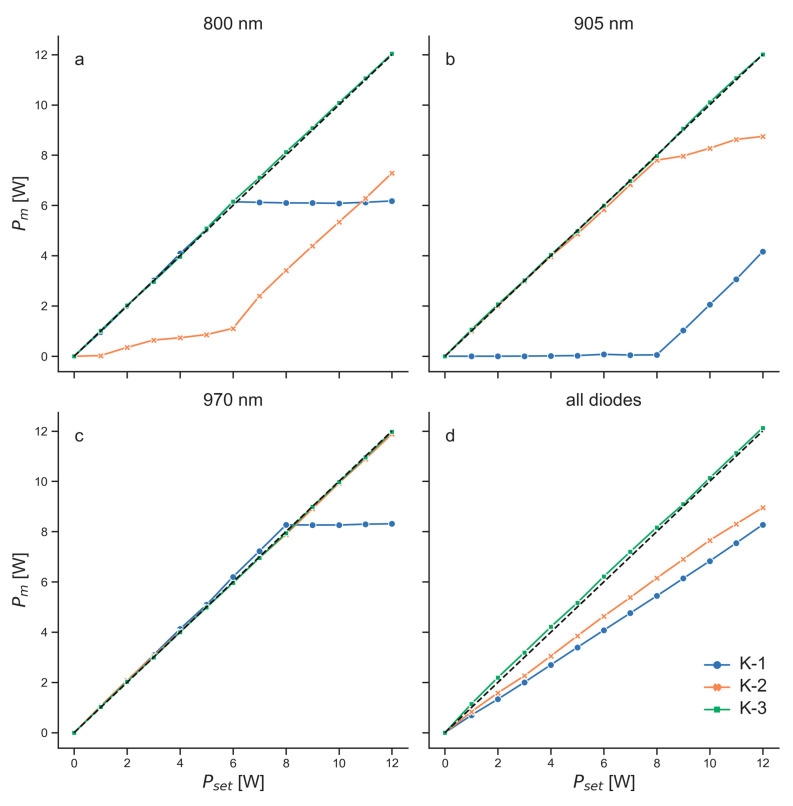
Power measurements of the three K-lasers (K-1, blue dots and blue lines; K-2, orange dots and orange lines; K-3, green dots and green lines) used as continuous wave (CW) lasers. Measured power ($P_{m}$) for different powers set at the devices ($P_{set}$). (**a**–**c**) Measurements using the three wavelengths (800 nm, 905 nm, 970 nm) individually; (**d**) measurements using all diodes simultaneously. Black dashed lines illustrate ideal curves where $P_{m}$ equals $P_{set}$.

Significant differences between $P_{m}$ and $P_{set}$ were observed for some laser diodes of the three K-laser units. Figure 4 shows measurements of the K-lasers used in CW mode. Two of the three investigated units had laser diodes that emitted much less power than expected from the settings. Specifically, the 905 nm diode of K-1 did not emit any substantial power (less than 0.1 W) for the first 8 W. Only when setting the power to 9 W and above, $P_{m}$ increased. For the other two diodes of K-1, a saturation effect was observed that started at 6 W (800 nm diode) and at 8 W (970 nm diode). When $P_{set}$ was increased above these values, $P_{m}$ did not increase further. A similar effect was seen for the 905 nm diode of K-2. This unit also showed a substantial problem with the 800 nm diode that was comparable to the 905 nm diode of K-1. Only K-3 emitted approximately the power that was expected from the settings for all three wavelengths. Using all diodes simultaneously, $P_{m}$ increased linearly with $P_{set}$ for both K-1 and K-2, although with a lower rate than expected. At the maximum power, they emitted 3.7 W (31%; K-1) and 3 W (25%; K-2) less than set. This performance can probably be assigned to the problems of their individual diodes. In contrast to the other two units, K-3, using all diodes, followed the expected curve relatively well. However, $P_{m}$ was between 0.13 W and 0.22 W larger than $P_{set}$ for this unit (1–15%).

**Figure 5:**
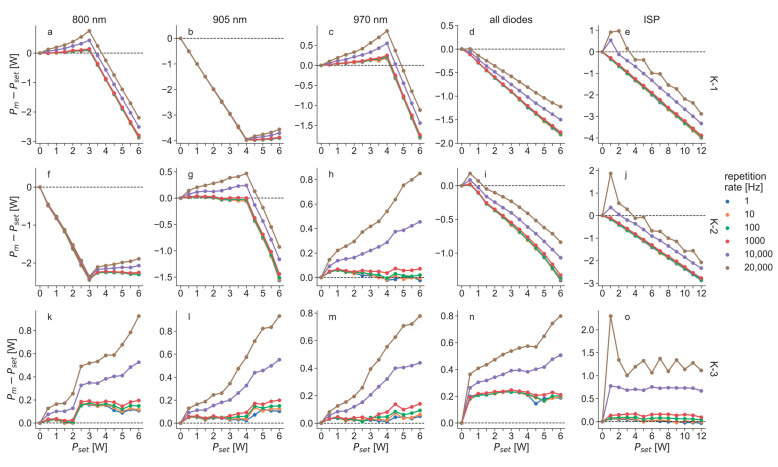
Difference between set powers ($P_{set}$) and measured powers ($P_{m}$) of the three K-lasers (K1, (**a**–**e**); K-2, (**f**–**j**); K-3, (**k**–**o**)) operated in pulsed wave (PW) mode at varying repetition rates. The lasers were measured using each wavelength individually: (**a**,**f**,**k**), 800 nm; (**b**,**g**,**l**), 905 nm; (**c**,**h**,**m**), 970 nm; they were also measured using all diodes simultaneously: (**d**,**i**,**n**), all diodes. In addition, they were measured in intense super pulse (ISP) mode, which is also using all diodes but in a different pulsing mode: (**e**,**j**,**o**), ISP. Dashed horizontal lines at zero in each diagram illustrate ideal curves where $P_{m}$ equals $P_{set}$.

In addition to the measurements in CW mode (Figure 4), operating the K-lasers with pulsed laser beams showed that increasing the repetition rate led to even stronger deviations between the set and the measured power (Figure 5). Specifically, measured powers at large repetition rates were significantly higher than at low repetition rates or at CW mode. This was observed for all three K-laser units and using all different modes. It should be noted that the positive deviations in Figure 5 correspond to a $P_{m}$ that was larger than $P_{set}$, i.e., the devices in such cases emitted more power than they were set to. Even the diodes that operated well in CW mode (K-2, 970 nm; K-3: all three diodes) showed values of $P_{m}$ that were well above $P_{set}$.

All three K-laser units emitted significantly more power than set when operated in ISP mode at low power (Figure 5). Especially when using high repetition rates, the K-lasers emitted up to three times the amount of power than expected ($P_{m}$ at $P_{set}$ = 1 W and 20 kHz: K-1, 1.91 W; K-2, 2.87 W; K-3, 3.30 W). Since this was observed for all three units, this is most likely a general problem caused by the laser diodes’ control mechanisms. When increasing the power, K-1 and K-2 deviated to lower values and emitted less power than they were set to, which is similar to their behavior in CW mode using all diodes (Figure 4d). K-3 followed the expected curve but consistently emitted too much power, especially when operated with high repetition rates.

**Figure 6:**
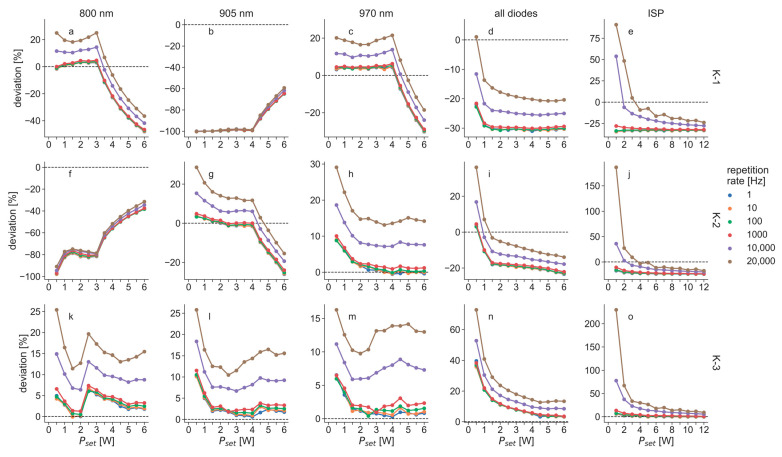
Relative deviation of measured power compared to set power as a percentage for the three K-lasers (K-1, (**a**–**e**); K-2, (**f**–**j**); K-3, (**k**–**o**)) operated in pulsed wave mode at varying repetition rates. Values were computed from measured power values that are shown in Figure 5. Dashed horizontal lines at zero in each diagram illustrate ideal curves.

Figure 6 shows the relative deviation of $P_{m}$ compared to $P_{set}$ for the K-lasers in PW mode, which were computed based on the measured power values shown in Figure 5. All three K-lasers emitted more than 20% or less than 20% of the powers that were set at the devices in at least one of the investigated settings, except for the 970 nm diode of K-3. The largest deviation of this diode was 16.3% for a repetition rate of 20 kHz and $P_{set}$ = 0.5 W. The other two laser diodes of K-3 emitted more than 20% too much power at the same settings and stayed within 20% for the rest. Using all diodes simultaneously, the deviations of K-3 were even greater and gave the largest deviation of all investigated modes: in ISP mode with $P_{set}$ = 1 W, and at a 20 kHz repetition rate, the deviation of K-3 was 230%.

### 3.3. K-Laser Pulse Measurements

**Figure 7:**
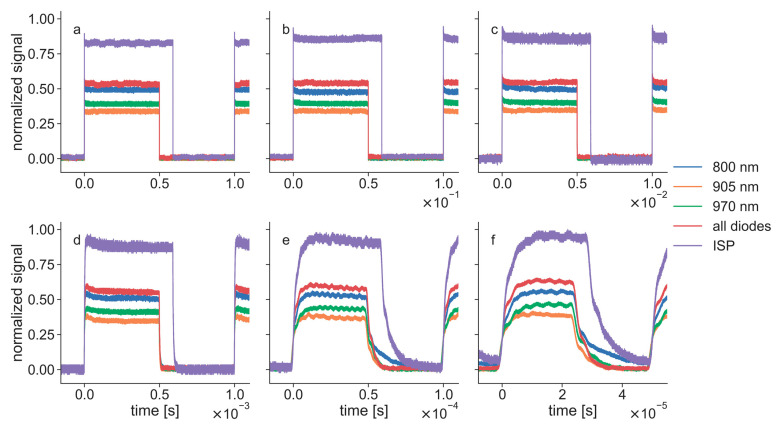
Light intensity in time for K-1 in the pulsed wave (PW) mode at different repetition rates: (**a**) 1 Hz; (**b**) 10 Hz; (**c**) 100 Hz; (**d**) 1 kHz; (**e**) 10 kHz; (**f**) 20 kHz. Pulses were recorded using the three diodes (800 nm, 905 nm, 970 nm) individually, all diodes simultaneously and using the K-lasers’ intense super pulse (ISP) mode. All measurements were done with K-1 set to maximum average power. The signals were normalized to the maximum value of each repetition rate.

Pulse measurements of the K-lasers operated in PW mode showed that the laser diodes in normal PW mode were modulated with a square-wave signal (Figure 7). The diodes were switched by the software on for half of a period and off for the other half cycle (i.e., 50% duty cycle). At high repetition rates, it was observed that this form of square-wave modulation was not adequately controlling the diodes since they could not be turned on or off instantaneously. The time to switch the diodes on (rise-time) and off (fall-time) could be computed best from the measurements at 20 kHz (rise-time, approximately 10 µs; fall-time, approximately 20 µs). In addition, the signals of the laser pulses had a short intensity peak at the beginning of a pulse (Figure 7). This peak was not observed for a repetition rate of 20 kHz since its length was on the same timescale as the pulse length at this repetition rate. Figure 7 shows recordings of K-1; K-2 and K-3 showed very similar behavior in the temporal domain. The differences in amplitude between the diodes in Figure 7 fit the differences in the average power of K-1 that were shown in Figure 5.

The pulses of the ISP mode showed that the amplitudes of these laser beams were larger than the amplitudes of normal mode signals, and the modulation was given by a rectangular wave instead of a square wave (Figure 7). In addition, the pulse lengths of the ISP signals were larger than the pulse lengths in normal mode. Therefore, the beams in ISP mode had a duty cycle that was larger than 50%. In a recent study with the same LTDs, it was shown that the pulse length of the K-lasers’ ISP mode signals depends on $P_{set}$ [7]. The temporal profiles shown in Figure 7 were measured with the ISP mode set to 12 W average power, which led to the maximum pulse length.

### 3.4. K-Laser Spatial Beam Characteristics

**Figure 8:**
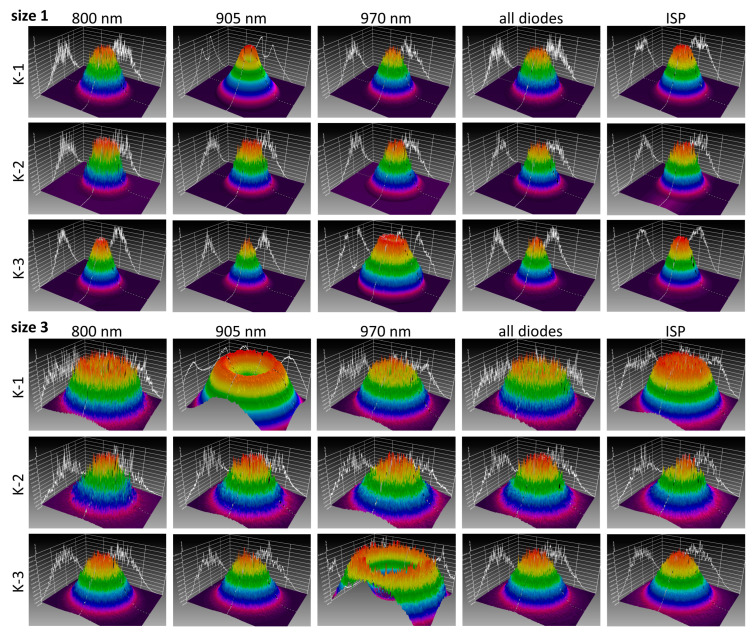
Color-coded light intensity distributions of the three K-lasers: for each unit, the three diodes (800 nm, 905 nm, 970 nm) were recorded individually as well as all diodes simultaneously and the K-lasers’ intense-super pulse (ISP) mode. Magenta represents the lowest light intensity, and red represents the highest light intensity. Projections of beam profiles along the horizontal and vertical axes are shown as white lines behind the intensity distributions. Recordings were taken with the K-lasers’ zoom objective set to sizes 1 and 3. The intensities of each recording were maximized by adjusting the camera exposure time.

Spatial intensity distributions of the K-laser units were recorded with the devices set to different beam sizes (Figure 8). The handpiece of the K-lasers had a zoom objective with a scale ranging from 1 to 5. According to the user manual, this translates to a beam area of 1 to 5 cm². However, since the beams were diverging, the beam area depended strongly on the distance between the handpiece and the recorder. This distance was kept to a minimum for the camera measurements (3 mm between the handpiece and the front of the camera). With this setup, most beam profiles were sufficiently small to be recordable with the camera, although the active area of the used camera sensor was only 1.25 cm^2^.

**Figure 9:**
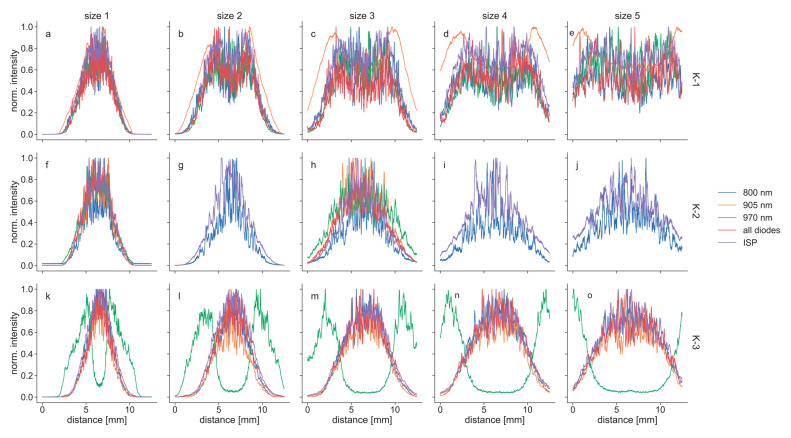
Beam profiles for the three K-lasers along a horizontal line across the intensity distributions: (**a**–**e**) K-1; (**f**–**j**) K-2; (**k**–**o**) K-3. The profiles were extracted from camera recordings of the individual diodes (800 nm, 905 nm, 970 nm), all diodes simultaneously and the K-lasers’ intense-super-pulse (ISP) mode. The sizes 1–5 were set at the zoom objective of each device. For K-2, four modes (800 nm, 905 nm, 970 nm, all diodes) were only recorded for sizes 1 and 3. All signals were normalized to their maximum intensity.

Most of the tested modes of the K-lasers led to laser beams with a Gaussian or near-Gaussian spatial profile, with the maximum intensity in their center. However, there were two diodes with strongly distorted laser beams: the 905 nm diode of K-1 and the 970 nm diode of K-3 had an intensity minimum at their center, which is best visible in the recordings at size 3 (Figure 8). This donut-like shape can also be seen in the beam profiles, which show the intensity along a horizontal line across the intensity distributions (Figure 9). From the modes of K-1, the 905 nm diode had the largest distortions and clearest donut shape, but all modes of this unit showed distorted beam profiles when the beam widths were increased, i.e., they all had a local intensity minimum in their center (Figure 9). This contrasts with K-3, where the 970 nm-diode was the only mode with a donut-shaped profile, whereas the remaining profiles were Gaussian or near-Gaussian. K-2 had no donut-shaped beams but showed relatively high noise levels compared to the Gaussian profiles of K-3.

## 4. Discussion

Two different LTDs (EMS laser and K-laser) were investigated by measuring multiple parameters of the laser beams emitted by each device. Three units of each LTD were investigated to differentiate differences between the set and measured light emission that were either due to malfunctions of individual units or pointing to potential, intrinsic problems of the instrument design of these devices. The EMS laser showed only small deviations between what was set at the device and the emitted laser beam. All three units performed similarly in the measured laser beam characteristics. In contrast, the K-laser units revealed substantial differences between what was set at the device and the measured emitted power. While some of these problems were seen only for individual laser diodes of the K-lasers, there were also some deficiencies that seemed to be general issues of this device.

The EMS lasers showed a maximum difference of 3.3% between the set and the actual power (Figure 2). The user manual of the EMS laser did not specify any errors for the emitted power. One unit emitted less power than set, which could be due to an unclear lens, micro-fractures in the fiber optic cable that transmits the light to the handpiece, or other alterations that can occur with such a device over time. The other two units emitted more power than that set, which might be a sign of minor misfits in the control mechanisms of the device. The EMS laser could only be operated in PW mode with the repetition rate as the only changeable parameter. The emitted light pulses should, therefore, all have identical amplitude and length. The pulse measurements showed that while the pulse lengths indeed stayed constant, the amplitude decreased with increasing repetition rate (Figure 3). Since two of the three investigated EMS laser units did not show a similar decrease in power for larger repetition rates, this does not seem to be affecting the overall light output. The camera recordings showed that all three units had comparable flat-top beam shapes, whereas the beam width differed (Figure 3). This might also be a sign of alteration, e.g., an unclear lens that would lead to light scattering. Overall, the differences between the units should have only little effect on the performance of the EMS laser in therapy.

For the K-laser, there were substantial differences between what was set at the device and the measured emitted power. There were dysfunctional laser diodes in two of the three investigated units of the K-laser (Figure 4). Since one of the K-lasers performed well in the power measurements of the CW mode with all three laser diodes (K-3), the dysfunctional laser diodes of K-1 and K-2 were signs of deterioration. These laser diodes emitted significantly less power than what was expected from the settings of the device. No sound technical explanation for the behavior of these laser diodes was found. It might have been due to broken laser diodes, damage to the fiber optic cable or other optical components, or misalignments of the laser diodes with respect to the optical beam path within the instrument. The measurements shown in the present study do not lead to a conclusive explanation. For example, the 905 nm diode of K-1, which emitted far too less power, also showed a highly distorted intensity distribution in the camera recordings (Figure 8). However, the 970 nm diode of K-3 also showed a similar donut-shaped intensity distribution but only very small deviations in the power measurements. The intensity distribution, therefore, cannot explain the deviations in power. The measured power of some laser diodes of K-1 and K-2 showed saturations at a larger $P_{set}$ (Figure 4). A misalignment of optical components in the beam path of these laser diodes could have led to this saturation, as a part of the light emitted by these laser diodes might not have ended in the fiber optic cable.

According to the user manual of the K-laser, the maximum average power of the device is 15 W. In the measurements for the present study; however, this was only possible when selecting all diodes simultaneously. With a single diode selected, the maximum average power was 12 W. Since the laser diodes were measured individually for this study, 12 W was the maximum average power that was tested. Furthermore, according to the user manual of the K-laser, the power emissions in CW mode can have an error of ±20%; the user manual did not specify potential errors for the power in PW or ISP mode. From the three units of the K-laser tested, only K-3 was within ±20% of the set power in CW mode. In PW mode, the measured power increased with larger repetition rates for all tested laser diodes of all three K-laser units. Only one of the tested K-laser diodes stayed within ±20% of the set power during all investigated settings (970 nm diode of K-3, see Figure 6). All other laser diodes were above 20% for some settings. This shows that none of the K-laser units tested was operating fully as was expected from the settings, which supports the hypothesis of a general problem with the laser diodes’ current control and their modulation.

The laser diodes that showed problems in CW mode showed similar behavior in the power measurements of the PW mode. Additionally, they emitted increasing power for larger repetition rates. The laser diodes of the K-lasers that gave a good performance in CW mode also emitted more power when operated at large repetition rates. This increase in the deviation can be explained by the discrepancy between the rise and fall times of the laser diodes and their modulation, which was observed in the temporal profiles (Figure 7). The laser diodes in normal PW mode were driven with a square-wave modulation. An ideal laser diode would turn on and off instantaneously. However, a real diode needs some time to be switched on and off and can, therefore, never match a square-wave signal perfectly. This becomes problematic when the rise and fall times are on the same time scale as the pulse length of a laser beam. For the laser diodes of the K-laser, the fall time was approximately 20 µs, and the rise time was approximately 10 µs. At 20 kHz, the pulse length of a laser beam with a 50% duty cycle was 25 µs. Therefore, the observed rise and fall times significantly affected the laser pulse forms when the repetition rates were in the kilohertz range. Since the fall time was larger than the rise time, as can be seen in Figure 7, the diodes effectively emitted more power than with an ideal square wave modulation. This explains why the deviations increased for all laser diodes for large repetition rates (Figure 5 and Figure 6). The overmodulation of the laser diodes of the K-laser was observed in all three tested units and might therefore be a general problem of this particular device.

Overall, all three K-lasers that were investigated in the present study showed two types of problems. First, there were dysfunctional laser diodes in two of the three investigated units that led to emitted power values that were smaller than the output expected from the settings. Secondly, all three units operated with a modulation that led to too much emitted power at large repetition rates (up to 230% of the expected value). The described problems cannot be generalized to other LTDs that were not measured for the present study. However, whether or not an LTD is operating correctly cannot be assessed by the typical user. The problems were detected only with professional, high-fidelity laser measurement equipment (Table 1) in a laboratory environment.

In general, malfunctioning LTDs can lead to mistreatment of patients that both the therapist and the patient would not notice. Additionally, it can have implications for the significance of clinical studies. Considering that laser therapy is currently debated for the treatment of a variety of medical conditions [1,2,3,4,5,6] and that it remains controversial (e.g., [9,10]), it is highly important that the LTDs used in clinical trials can be trusted. An LTD from the K-laser Cube series was used in at least three clinical studies [11,12,13] in which the light output was not measured. A positive outcome after laser treatment was reported in one of these studies [11]. The second study reported some clinical improvement but no effect of the treatment [12]. The third trial had to be aborted because of unexpected adverse effects that were potentially linked to the laser treatment [13]. Of note, at present, there is no evidence that the deviations to larger emitted power found in the present study are harmful or actually caused unwanted side effects when using the K-laser units investigated in the present study in clinical settings. This would need to be investigated in further studies.

Finally, a recent study from Brazil should be mentioned, in which certain laser beam properties (average power and laser beam diameter) of multiple units of three different LTDs operating at wavelengths between 450 nm and 904 nm were investigated [8]. Compared with the values expected from the user manuals of these LTDs, the measured average power varied between 2% and 134% and the laser beam diameter between 38% to 543%. However, the average measured optical power emission of these devices was between 0.61 and 103 mW and, thus, more than a power of ten lower than the average optical power emission of the LTDs investigated in the present study.

## 5. Conclusions

In summary, together with the results of the aforementioned study performed in Brazil [8], the results of the present study indicate the need for better standardization and consistency of laser light sources used in medicine.

## Figures and Tables

**Table 1:** Sensors for laser beam characterization used in the present study, together with their equipment and selected sensitivity measures.

Type	Model	Spectral Range [nm]	Range/Bandwidth	Sensitivity Measure	Sensitivity Value
Thermal power sensor	50(150)A-BB-26-PPS (Ophir)	190–2000	40 mW–150 W	Power noise level	2 mW
Photodiode	FPD-VIS300 (Ophir)	320–1100	>1.2 GHz	Rise/fall time	<0.3 ns
Oscilloscope	HMO3524 (Hameg) *	-	350 MHz	Sampling rate	4 GSa/s
Oscilloscope	MSO7024 (Rigol)	-	200 MHz	Sampling rate	10 GSa/s
Optical diffusor	DG20-220-MD (Thorlabs)	350–2000	-	-	-
Beam profiling camera	LT665 (Ophir)	190–1100	54 dB	Pixel size	4.54 µm

* The HMO3574 was used to record pulses from EMS-1 and K-2. Abbreviations: nm, nanometers; W, Watt; nanoseconds, ns; GS/s: Gigasamples per second; decibels, dB.

## Data Availability

The data presented in this study are openly available in Zenodo at doi.org/10.5281/zenodo.7525417, reference number [14].
